# Ticagrelor or clopidogrel dual antiplatelet therapy following a pharmacoinvasive strategy in ST‐segment elevation myocardial infarction

**DOI:** 10.1002/clc.23716

**Published:** 2021-08-18

**Authors:** Robert C. Welsh, Jay S. Shavadia, Yinggan Zheng, Benjamin D. Tyrrell, Raymond Leung, Kevin R. Bainey

**Affiliations:** ^1^ Canadian VIGOUR Centre University of Alberta Edmonton Alberta Canada; ^2^ Division of Cardiology, Department of Medicine University of Alberta Edmonton Alberta Canada; ^3^ Cardiac Sciences Mazankowski Alberta Heart Institute Edmonton Alberta Canada; ^4^ Division of Cardiology, Department of Medicine University of Saskatchewan Saskatoon Saskatchewan Canada; ^5^ Cardiology CK Hui Heart Centre Edmonton Alberta Canada

**Keywords:** antiplatelets, pharmacoinvasive reperfusion, fibrinolysis, STEMI

## Abstract

**Objectives:**

To describe and evaluate outcomes in STEMI patients sustained on clopidogrel compared to those switched to ticagrelor following fibrinolysis.

**Background:**

World‐wide, many STEMI patients cannot achieve timely PCI and therefore require fibrinolysis. Although comparable 30‐day and 1‐year safety was shown with clopidogrel or ticagrelor in the TREAT study, there is paucity of long‐term outcomes in pharmacoinvasive treated STEMI.

**Methods:**

We conducted an observational cohort study evaluating consecutive pharmacoinvasive STEMI patients treated in a network, comparing those switched to ticagrelor to those sustained on clopidogrel. The primary efficacy composite was one‐year all‐cause death, recurrent myocardial infarction, and stroke with major bleeding and intracranial hemorrhage (ICH) as the safety outcomes. Multivariable Cox regression model was used to examine the association between P2Y12 inhibitor and outcomes with inverse probability weighting.

**Results:**

Of 1426 pharmacoinvasive STEMI patients, 28% (*n* = 396) were converted to ticagrelor at a mean of 9.9 h after fibrinolysis with comparable GRACE Risk Scores (median; 158 vs 157, p0.352). The primary composite occurred in 3.5% of ticagrelor and 7.0% of clopidogrel treated patients (p0.014). Following adjustment, ticagrelor was associated with a 54% lower composite outcome (adjusted HR 0.46, 95% confidence interval 0.26–0.84). Major bleeding 6.3% vs 6.1% (NS) and ICH 0.0% vs 0.2% (NS) were similar.

**Conclusions:**

In a prospective STEMI cohort, switching to ticagrelor compared with sustaining clopidogrel following fibrinolysis pharmacoinvasive reperfusion reduced recurrent ischemic events at 1‐year with no differences in major bleeding or ICH. Aligned with randomized data, these findings provide support to switch pharmaco‐invasively treated STEMI patients.

## 1 INTRODUCTION

The prognostic benefit associated with dual antiplatelet therapy (DAPT) following acute coronary syndromes (ACS) has been well established.^1^, ^2^ However, the persistent risk of recurrent ischemia following the index ACS presentation on traditional clopidogrel based DAPT has led to the need to intensify existing secondary prevention therapies. As such, newer and more potent oral P2Y12 antagonists‐prasugrel and ticagrelor‐have been preferentially endorsed over clopidogrel in ST elevation myocardial infarction (STEMI) patients following primary percutaneous coronary intervention (PCI).^3^, ^4^, ^5^

Randomized trials that demonstrated superior efficacy of ticagrelor and prasugrel however excluded STEMI patients treated with a contemporary fibrinolytic pharmaco‐invasive strategy.^6^, ^7^, ^8^, ^9^ As such, in the absence of high‐quality data, guidelines continue to recommend clopidogrel as the agent of choice for STEMI patients receiving pharmacological reperfusion although there is acknowledgement that switching from clopidogrel to a more potent P2Y12 antagonist seems reasonable once the patient has stabilized (48 h).^3^, ^4^, ^5^ Clinical practice guidelines however provide no recommendations on whether to switch clopidogrel for a more potent P2Y12 antagonist after cardiac catheterization in this sub‐group of patients. Intuitively, the observed efficacy benefit associated with the more potent P2Y12 antagonists with primary PCI should be translated to pharmaco‐invasively treated patients, however, the increase in TIMI major non‐CABG bleeding observed within PLATO^7^ and TRITION TIMI 38^6^ trials raises potential safety concerns with switching early after fibrinolysis.

The ticagrelor in patients With ST‐elevation myocardial infarction treated with pharmacological thrombolysis (TREAT) study demonstrated the safety of switching from clopidogrel to ticagrelor within 24 h following fibrinolysis which was consistent at 30 days and 1 year.^10^, ^11^ Our objective is to describe the pattern of in‐hospital P2Y12 antagonist switching following fibrinolysis and second to evaluate associated clinical outcomes of pharmaco‐invasively treated STEMI being discharged on ticagrelor compared to clopidogrel within a comprehensive STEMI network of care.

## 2 METHODS

### 2.1 Vital heart response

The vital heart response (VHR) program is a regional reperfusion network of care, developed in 2005 (involving all admitting hospitals with a referral population of approximately 2 000 000 inhabitants), to implement timely, and evidence‐based reperfusion therapies to maximize the outcomes of STEMI patients in Central and Northern Alberta. In brief, a 24‐h on call VHR cardiologist co‐ordinates care between the pre‐hospital emergency medical services or physicians in non‐PCI capable hospital emergency rooms, and based on the clinical scenario, an electronically transmitted ECG, and estimated timings of transfer, decides on 1 of the 2 reperfusion options (pharmacoinvasive or primary PCI).^12^ In the pharmacoinvasive (PI) strategy which has been consistently applied since VHR inception, bolus weight‐based tenecteplase (TNK), aspirin, clopidogrel and enoxaparin is administered according to guideline recommendations and followed by either rescue PCI or scheduled angiography (within 6–24 h) consistent with the STrategic Reperfusion Early after Myocardial infarction study.^9^ This includes ½ dose TNK for patient >75 years of age. The need for rescue PCI is determined according to reperfusion success measured by <50% ST segment resolution in the ECG lead with the maximal ST‐elevation 60–90 min after TNK bolus, hemodynamic instability or refractory ventricular arrhythmias. In successfully reperfused STEMI, scheduled catheterization is performed 6–24 h after the administration of TNK. The choice of P2Y12 receptor antagonist at hospital discharge in pharmaco‐invasively treated patients is left to the discretion of the managing cardiologist. Those patients converted to ticagrelor received a 180 mg oral loading dose at the time of switch. Prasugrel is not utilized within our STEMI network.

Consecutive STEMI hospitalizations were recorded as part of a comprehensive and inclusive VHR Registry. A standard definition of STEMI was utilized and determined by adjudication of the electrocardiogram (ECG) by VHR cardiologists. The registry contains detailed clinical information obtained by chart review including patient demographics, medical history, hospitalization characteristics, in‐hospital procedures and pharmacotherapy, and in‐hospital clinical events. Data collection are conducted by a trained analyst using standardized definitions. Post‐discharge clinical events within 1 year were obtained via the Alberta SPOR SUPPORT Unit, a jointly funded program by Alberta Innovates and the Canadian Institutes of Health Research to support patient‐oriented research. ICD‐10 codes were used to define those events in provincial health databases and follow‐up status from the Alberta Health Care Insurance Plan (AHCIP) registry. Those events included all‐cause death, recurrent myocardial infarction (MI), stroke, major bleeding and intracranial hemorrhage (Table S1). The study cohort timeframe was from January 2013 to March 2017.

### 2.2 Definition of end‐points

The primary efficacy endpoint was defined as time to first event of a composite of all‐cause death, recurrent MI or stroke within 1 year from first medical contact (FMC) and primary safety endpoint was major bleeding within 1 year. Follow‐up was complete at index hospital discharge in all patients; between discharge and 1 year, 6.8% (*n* = 98) of patients either could not be linked with follow‐up data or they left the province. These patients were censored at either discharge or end of the last fiscal year in the AHCIP registry.

### 2.3 Statistical analysis

Continuous variables were reported as medians with 25th and 75th percentiles, whereas categorical variables were presented as count and percentages. Differences between groups (no switch versus switch) were tested using Wilcoxon rank‐sum test for continuous variables and the *χ* ^2^ test or Fisher's exact test for categorical variables.

**FIGURE 1:**
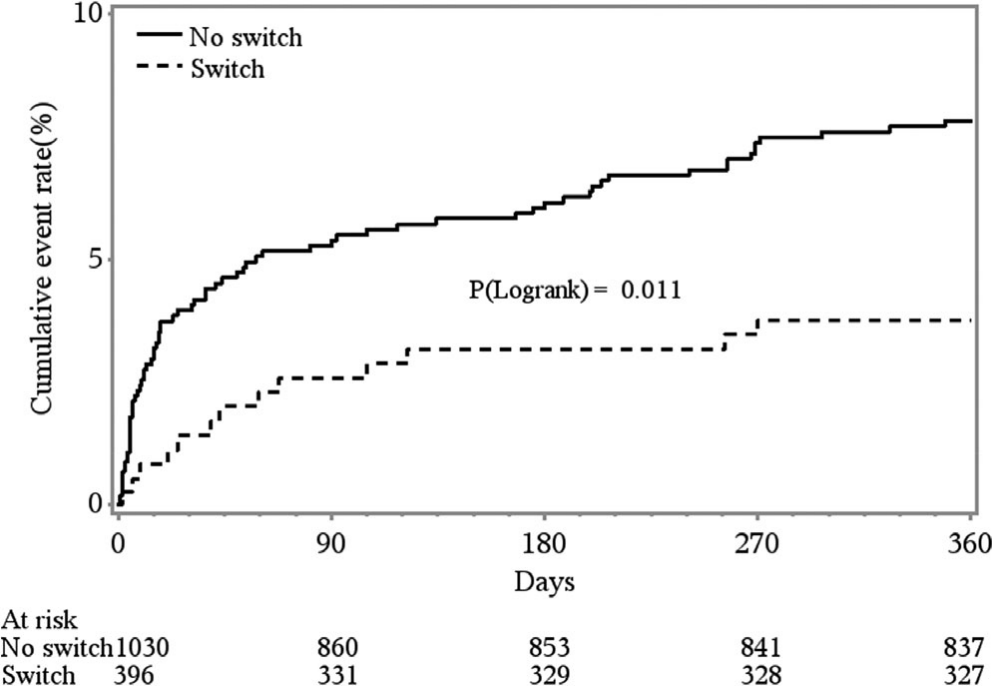
Kaplan–Meier estimated cumulative incidence of 1‐year all cause death, recurrent myocardial infarction or stroke according to antiplatelet therapy. Patients sustained on clopidogrel (no‐switch) are displayed in the solid line and patients switched to ticagrelor (switch) are displayed in the hashed line

Kaplan–Meier curves were used to display the unadjusted relationship between switch and the primary efficacy endpoint (i.e., the time to the first occurrence of all‐cause death, recurrent MI, or stroke within 1 year [Figure 1]) with comparison between groups using the log‐rank test. Kaplan–Meier estimated rates and 95%CI within 1 year were also reported. The relative association between switch and primary efficacy endpoint and association between switch and primary safety endpoint were examined using Cox proportional hazard regression. Unadjusted and adjusted hazard ratios (HR) and 95% confidence intervals (CI) were reported.

### 2.4 Inverse probability weighting adjusted analysis

To account for selection bias and confounders, the association between switch and primary efficacy endpoint and association between switch and primary safety endpoint were adjusted using an inverse probability weighting (IPW) approach. For this IPW analysis, a propensity score model for switch versus no switch was first developed using logistic regression. Patient characteristics available at FMC (defined as ambulance arrival or hospital admission if the patient self‐presented) such as age, sex, history of diabetes, history of hypertension, history of angina, prior MI, family history of coronary artery disease (CAD), history of hypercholesterolemia, history of stroke, history of heart failure, history of atrial fibrillation, systolic blood pressure, heart rate, body mass index, current smoker, and time from symptom onset to FMC were forced into the propensity score model. Using the inverse of the derived propensity score for switch versus no switch as weights, a Cox proportional hazards model was then applied to evaluate the adjusted association between switch and primary efficacy endpoint and association between switch and primary safety endpoint. A restricted cubic spline function was used to test the linearity assumption for continuous variables in the model. Spline transformations were applied when linearity assumption was violated. Acknowledging the potential confounding influence of the need for oral anticoagulation or coronary artery bypass grafting (CABG) on clinician P2Y12 selection, two sensitivity analyses were performed by (1) excluding patients receiving oral anticoagulants at hospital discharge; and (2) excluding patients who underwent CABG using the same method described above.

A descriptive analysis was conducted assessing the primary efficacy and safety outcome according to quartiles of time following fibrinolysis that ticagrelor was administered (*n* = 344). The Cochran‐Armitage trend test was used to test whether the frequency of efficacy and safety endpoints differed across the quartiles of time.

All statistical tests were two‐sided with *p*‐value <0.05 considered as statistically significant. Statistical analyses were performed using the SAS (version 9.4; Cary, NC). No correction was made for multiple comparison. The study was approved by the University of Alberta Ethics Review Board with individual consent waived because of privacy rules related to this quality assurance registry.

## 3 RESULTS

Of the 1426 STEMI patients receiving a pharmacoinvasive reperfusion strategy between 2013 and 2017, 1030 were sustained on clopidogrel while 396 were switched in‐hospital to ticagrelor. The proportion of patients switched to ticagrelor increased each year with 13.9% in 2013, 25.4% in 2014, 30.5% in 2016, and 60.4% in 2017 of the total annual pharmacoinvasive STEMI population. Baseline characteristics and comorbid medical conditions at hospital admission are depicted in Table 1. It was less likely to switch to ticagrelor in patients with pre‐existing atrial fibrillation (ticagrelor 0.5% versus clopidogrel 3.1%, *p* = 0.004) with the remainder of the variables similar.

**TABLE 1:** Selected baseline characteristics according to switch

	All patient (*n* = 1426)	No switch (*n* = 1030)	Switch (*n* = 396)	*p* ^a^
Age, years	58 (51, 65)	58 (51, 65)	58 (51, 65)	0.68
Female, *n* (%)	274 (19.2)	200 (19.4)	74 (18.7)	0.75
Weight, kg	88 (77, 100)	87 (76, 100)	88 (77, 100)	0.76
BMI, kg/m^2^	29 (26, 33)	29 (26, 33)	29 (26, 33)	0.93
Heart rate, beats per minute	75 (63, 86)	75 (64, 86)	75 (61, 86)	0.71
Systolic BP, mmHg	140 (125, 159)	140 (126, 159)	142 (124, 160)	0.67
Diastolic BP, mmHg	89 (76, 101)	89 (76, 101)	89 (77, 103)	0.25
Comorbidities, *n* (%)				
Hypercholesterolemia	515 (36.1)	358 (34.8)	157 (39.6)	0.085
Hypertension	656 (46.0)	469 (45.5)	187 (47.2)	0.57
Diabetes	265 (18.6)	184 (17.9)	81 (20.5)	0.26
Current smoker	770 (54.0)	570 (55.3)	200 (50.5)	0.10
Family history of CAD	301 (21.1)	226 (21.9)	75 (18.9)	0.21
Medical History, *n* (%)				
Coronary artery disease	78 (5.5)	56 (5.4)	22 (5.6)	0.93
Myocardial infarction	221 (15.5)	158 (15.3)	63 (15.9)	0.79
Coronary revascularization	200 (14.0)	135 (13.1)	65 (16.4)	0.11
Stroke	38 (2.7)	25 (2.4)	13 (3.3)	0.37
Heart failure	14 (1.0)	11 (1.1)	3 (0.8)	0.59
Atrial fibrillation	34 (2.4)	32 (3.1)	2 (0.5)	0.004
Time intervals				
Time–Sx to FMC, min	61 (28, 144)	61 (30, 142)	62 (28, 157)	0.96
Time–FMC to TNK, min	46 (29, 88)	45 (29, 86)	47 (29, 92)	0.50
Time–TNK to ticagrelor, h	9.9 (2.8, 33.1)	—	9.9 (2.8, 33.1)	NA
Length of hospital stay, day	5 (4, 6)	5 (4, 6)	5 (4, 6)	0.72
In‐hospital procedures, *n* (%)				
Glycoprotein IIb/IIIa inhibitor	241 (16.9%)	80 (20.2%)	161 (15.6%)	0.039
Angiogram alone (no PCI)	163 (11.4)	137 (13.3)	26 (6.6)	<0.001
Rescue PCI	451 (31.6)	303 (29.4)	148 (37.4)	0.004
Urgent PCI	482 (33.8)	359 (34.9)	123 (31.1)	0.175
Elective PCI	406 (28.5)	274 (26.6)	132 (33.3)	0.012
CABG	33 (2.3)	30 (2.9)	3 (0.8)	0.015
Valve replacement	1 (0.1)	1 (0.1)	0 (0.0)	0.535
Pacemaker	7 (0.5)	5 (0.5)	2 (0.5)	0.962
Implantable cardiac defibrillator	1 (0.1)	0 (0.0)	1 (0.3)	0.107
In‐hospital events, *n* (%)				
Cardiac arrest	86 (6.0)	57 (5.5)	29 (7.3)	0.204
Shock	61 (4.3)	45 (4.4)	16 (4.0)	0.784
IABP	22 (1.5)	16 (1.6)	6 (1.5)	0.958
Inotropes	47 (3.3)	36 (3.5)	11 (2.8)	0.497
Stroke	15 (1.1)	13 (1.3)	2 (0.5)	0.210
CHF	132 (9.3)	99 (9.6)	33 (8.3)	0.456
Recurrent MI	9 (0.6)	8 (0.8)	1 (0.3)	0.263
ICH	5 (0.4)	5 (0.5)	0 (0.0)	0.165
Major bleeding	73 (5.1)	54 (5.2)	19 (4.8)	0.733

*Note*: Continuous variables are expressed as median (25th‐75th percentiles); categorical variables expressed as frequencies and percentages.

Abbreviations: BMI, body mass index; BP, blood pressure; CABG: coronary artery bypass grafting; CHF, congestive heart failure; FMC, first medical contact; IABP, intra‐aortic balloon pump; ICH, intracranial hemorrhage; MI, myocardial infarction; PCI, percutaneous coronary intervention; Sx, symptom; TNK, tenecteplase.

^a^ Comparison between no switch and switch groups.

The time from symptom onset to first medical contact was approximately 1 hour with fibrinolysis administered 46 min there after (25% and 75% interquartile range (IQR) (29–88) min) (Table 1). In those patients switched to ticagrelor the mean time was 9.9 h after fibrinolysis with a broad range (25% and 75% IQR [2.8–33.1] h). Patients that were switched were more likely to have received percutaneous coronary revascularization (PCI) including in the rescue and scheduled angiography situations but were less likely to undergo coronary artery bypass grafting. In hospital clinical events were similar between the two groups including no difference in bleeding (ticagrelor 4.8% versus clopidogrel 5.2%, *p* = NS).

At discharge from hospital, application of evidence based medical therapy was excellent with high utilization of aspirin (97%), angiotensin converting enzyme inhibitors or angiotensin receptor blockers (93%), beta blockers (94%), and cholesterol lowering medications (97%) (Table S2). The only between group difference was with oral anticoagulation being more common in those sustained on clopidogrel (ticagrelor 6.8% versus clopidogrel 14.6%, *p* < 0.001).

The 1‐year primary composite of all cause death, recurrent myocardial infarction, and stroke was 52% lower in patients switched to ticagrelor compared with those sustained on clopidogrel (ticagrelor 3.5% versus clopidogrel 7.3%, HR 0.48 (95% confidence intervals (CI) 0.27–0.85) *p* = 0.013) (Figure 1) (Table 2). Additionally, there was a reduction in recurrent myocardial infarction and trend to reduction in stroke as well as the double end point of death and recurrent myocardial infarction. There was no difference in major bleeding or intracranial hemorrhage at 1 year between those sustained on clopidogrel or switched to ticagrelor.

**TABLE 2:** Clinical events within 1‐year according to no switch and switch

	All		No switch		Switch		
	(*n* = 1426)		(*n* = 1030)		(*n* = 396)		
	Observed Rate, *n*(%)	KM rate %(95%CI)	Observed Rate, *n*(%)	KM rate %(95%CI)	Observed Rate, *n*(%)	KM rate %(95%CI)	*p* ^a^
Death/re‐MI/	89 (6.2)	7.0(5.7–8.5)	75 (7.3)	8.0(6.5–10.0)	14 (3.5)	4.1(2.4–6.8)	0.011
Stroke							
Death/re‐MI	67 (4.7)	5.3 (4.2–6.6)	55 (5.3)	5.9(4.6–7.6)	12 (3.0)	3.5 (2.0–6.0)	0.071
Death	24 (1.7)	2.0 (1.3–2.9)	18 (1.7)	2.0(1.3–3.2)	6 (1.5)	1.7 (0.8–3.8)	0.76
Re‐MI	44 (3.1)	3.4 (2.6–4.6)	38 (3.7)	4.1(3.0–5.6)	6 (1.5)	1.8 (0.8–3.9)	0.036
Stroke	27 (1.9)	2.2 (1.5–3.2)	24 (2.3)	2.7(1.8–4.0)	3 (0.8)	0.9 (0.3–2.8)	0.053
Major bleeding	88 (6.2)	6.2 (5.0–7.6)	63 (6.1)	6.1(4.8–7.8)	25 (6.3)	6.3 (4.3–9.2)	0.93
ICH	7 (0.5)	0.5 (0.3–1.1)	7 (0.7)	0.8(0.4–1.6)	0 (0.0)	0 (0–0)	0.10

^a^ Comparison between no switch and switch groups based on KM estimated rate (Log‐rank Chi‐square). ICH, intracranial hemorrhage; KM, estimated Kaplan Myer; MI, myocardial infarction.

**FIGURE 2:**
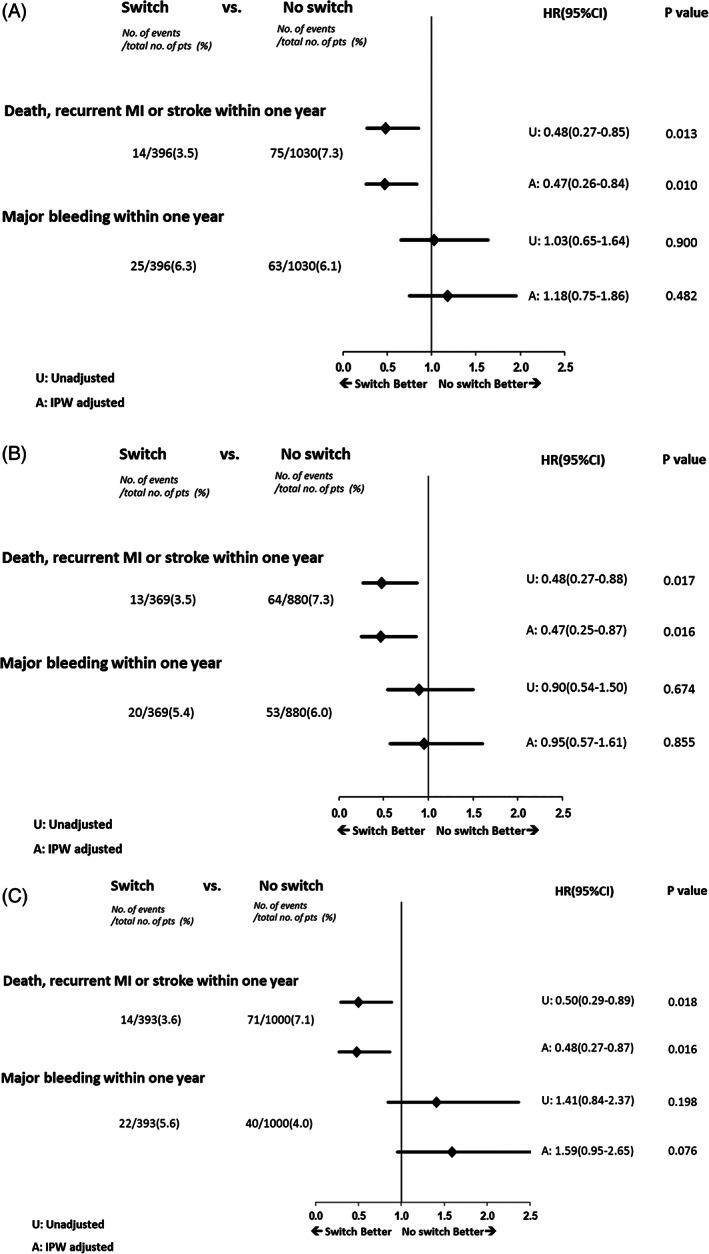
Association between switching to ticagrelor (switch) compared to sustaining clopidogrel (no switch) and time to first occurrence of all‐cause death, recurrent MI or stroke within 1 year. HR(95%CI) ‐ hazard ratio (95% confidence interval). (A) Association between switching to ticagrelor (switch) compared to sustaining clopidogrel (no switch) and time to first occurrence of all‐cause death, recurrent MI or stroke within 1 year after patients discharged with oral anticoagulants are excluded, *n* = 177. HR (95%CI) ‐ hazard ratio (95% confidence interval). (B) Association between switching to ticagrelor (switch) compared to sustaining clopidogrel (no switch) and time to first occurrence of all‐cause death, recurrent MI or stroke within 1 year after patients with in hospital CABG excluded, *n* = 33. HR (95%CI) ‐ hazard ratio (95% Confidence Interval), IPW ‐ inverse probability weighting

After IPW adjustment the one‐year composite primary endpoint of death, recurrent myocardial infarction and stroke remained reduced with a 53% reduction (hazard ratio 0.47 (95% CI [0.26–0.84], *p* = 0.01) (Figure 2). Further, the adjusted risk of major bleeding remained similar between groups (hazard ratio 1.18 [95% confidence intervals 0.75–1.86], *p* = 0.48). Acknowledging the potential impact of oral anticoagulants and CABG, the unadjusted and adjusted outcomes were assessed in patients on sustained clopidogrel or switched to ticagrelor after excluding those patients discharged on an oral anticoagulant and undergoing CABG in two sensitivity analyses. Both the enhanced efficacy and safety of switching to ticagrelor remained similar to the entire cohort for patients discharged on oral anticoagulants (Figure 2(A)). After excluding patients that had undergoing CABG, the enhanced efficacy was maintained consistent with the overall cohort with numerically increased bleeding associated with switching (adjusted HR 1.59, [95% CI 0.95–2.65], *p* = 0.076) (Figure 2(B)).

**FIGURE 3:**
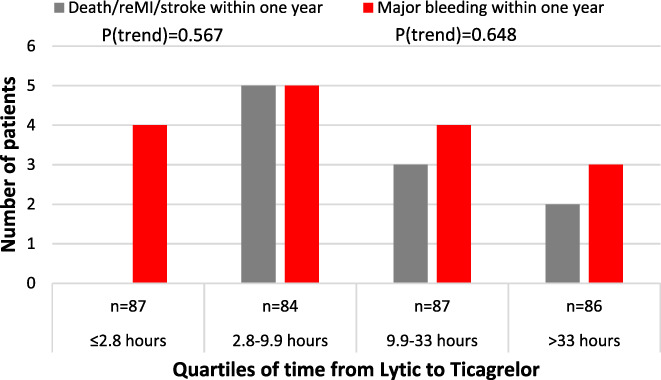
Outcomes within 1 year according to quartiles of time from receiving fibrinolysis to initial administration of ticagrelor

The cohort of patients that were switched to ticagrelor were divided into quartiles of time from fibrinolysis to assess the association of the timing of switching with clinical events. Acknowledging the reduced number of patients in each quartile, there was no association detected between the timing of switching and the composite ischemic outcome or major bleeding events (Figure 3).

## 4 DISCUSSION

Within a comprehensive regional STEMI network of care, this study provides insight into the clinical DAPT strategy in fibrinolysis pharmacoinvasive treated STEMI patients assessing outcomes in those patients sustained on clopidogrel or switched to ticagrelor. Over the observation period the frequency of switching from clopidogrel to ticagrelor increased approximately 10% yearly from 15% in 2013 to 60% in 2017. The in‐hospital ischemic and bleeding events were similar between those with sustained clopidogrel and switched to ticagrelor. However, the one‐year composite of death, recurrent myocardial infarction and stroke was decreased by 53% (adjusted HR 0.47, [95% CI 0.26–0.84], *p* = 0.016) without an increased risk of major bleeding (adjusted HR 1.18 [95% CI 0.75–1.86], *p* = 0.48) in patients on ticagrelor. These results were sustained when patients receiving oral anticoagulants at discharge were excluded with a consistent 53% reduction in the composite ischemic event (adjusted HR 0.47 [95% CI 0.25–0.87], *p* = 0.016) and no increased risk of major bleeding (adjusted HR 0.95 [95% CI 0.57–1.61], *p* = 0.86). When patients with in‐hospital CABG were excluded, there was a consistent 52% reduction in the composite ischemic event (adjusted HR 0.48 [95% CI 0.29–0.87], *p* = 0.016) with a non‐significant increase risk of major bleeding (adjusted HR 1.59, [95% CI 0.95–2.65], *p* = 0.76). Our observational data provides reassurance for clinicians to switch from clopidogrel to ticagrelor following a fibrinolysis pharmacoinvasive reperfusion strategy for STEMI including in those requiring rescue PCI for clinically failed reperfusion and in those undergoing scheduled PCI following successful reperfusion.

Previous studies have demonstrated that following an evidenced based fibrinolysis strategy with clopidogrel that approximately two thirds of patients have high platelet reactivity.^13^, ^14^ Further, these small studies with platelet reactivity endpoints demonstrate that ticagrelor is superior even to high‐dose clopidogrel.^13^, ^14^ Accordingly, the strategy of early switching from clopidogrel to ticagrelor at the time of PCI following fibrinolysis could be associated with lower risk of recurrent MI which is consistent with the findings in this analysis.

The ticagrelor in patients With ST‐elevation myocardial infarction treated with Pharmacological Thrombolysis (TREAT) trial was a multicentered international trial with STEMI patients <75 years of age randomized to sustained clopidogrel or ticagrelor following fibrinolysis. Approximately 40% of patients received fibrinolysis with tenectaplase with nearly 20% receiving non‐fibrin specific agents. There was variable application of contemporary pharmacoinvasive approach with only 56% of patient in TREAT received PCI during their index hospitalization. The current analysis was conducted in an integrated STEMI system of care that has applied a dedicated evidence based pharmacoinvasive approach utilizing tenecteplase since its inception. Indeed, in the current analysis, all patients underwent coronary angiography during the index hospitalization with 89% undergoing PCI with implantation of drug eluting stents. Furthermore, 32% of patients had rescue PCI following unsuccessful fibrinolysis with the need for rescue appearing to influence clinician's decision to switch to ticagrelor.

Moreover, in the TREAT study, the median time of randomization was 11.4 h (interquartile range, 5.8–18.2 h) after fibrinolysis with antiplatelet study medications administered thereafter. In the current analysis, the median time from fibrinolysis to switching to ticagrelor was less than 10 h with a broad range including one quarter of all patients switched within 3 h of fibrinolysis. Acknowledging the small sample of patients, there was no associated increased risk of major bleeding in patients switched early after fibrinolysis. While clinicians may feel more comfortable switching from clopidogrel to ticagrelor further from the time of fibrinolysis, such as at the time of scheduled angiography and PCI for those with successful pharmacological reperfusion, roughly one‐third of patients will require rescue or urgent intervention. Our data is useful in this context as we have demonstrated no difference in bleeding events when switched early, that is, for rescue PCI or urgent PCI. While provocative, our findings do require confirmation in an appropriately powered clinical trial – however in the absence of such a study, this novel finding may assist practicing clinicians in switching earlier to ticagrelor at the time of rescue or urgent PCI following fibrinolysis.

The current analysis demonstrates a similar pattern of clinical practice to the Platelet inhibition and Patient Outcomes (PLATO) study where ticagrelor was administered at full loading dose early after STEMI diagnosis in patients with prior clopidogrel therapy; although it is acknowledged that PLATO indeed excluded the exact patients, we have studied.^7^ In patients post stent implantation there is substantial interest in: (1) the optimal timing of potent antiplatelet administration, (2) the duration of dual antiplatelet therapy and (3) switching to less potent antiplatelet strategies to minimize bleeding risk and decrease costs.^15^, ^16^, ^17^, ^18^, ^19^ Although many guidelines still recommend 1 year of potent dual antiplatelet therapy following acute myocardial infarction, the recent TicAgrelor versus cLOpidogrel in Stabilized patients with Acute Myocardial Infarction (TALOS‐AMI) suggested sustained efficacy and enhanced safety converting from ticagrelor to clopidogrel based dual antiplatelet therapy at 1 month following myocardial infarction with stenting.^20^ In the future, head to head comparisons of the multiple antiplatelet strategies in a large scale appropriately powered megatrial would help guide clinical care.

### 4.1 Limitations

The current analysis is based on observational data and despite IPW adjustments for baseline variables there may be inherent patient related bleeding risk differences including patient age driving clinical decisions on the choice of the P2Y12 antagonist. In‐hospital procedures and medications were not adjusted since those characteristics may confounded with switching. Additionally, we did not evaluate the effects on patients who had ticagrelor switched to clopidogrel for any clinical indication. Accordingly, given the observational nature of this study, despite adjustment of clinical outcomes for baseline risk, significant confounding may exist.

## 5 CONCLUSION

In a large prospective STEMI registry, we found switching patients to ticagrelor compared with sustaining clopidogrel therapy following fibrinolysis pharmacoinvasive reperfusion was associated with reduced recurrent ischemic events at 1‐year. Additionally, switching was not associated with differences in major bleeding or ICH. These findings provide support for switching to ticagrelor in pharmacoinvasive reperfusion patients and add to randomized clinical trial results.

## CONFLICT OF INTEREST

Dr. Welsh reports grants and personal fees from Astra Zeneca, Bayer, and Boehringer Ingelheim outside the submitted work. Dr. Tyrrell reports personal fees from Astra Zeneca and Bayer. Dr. Bainey reports personal fees from Astra Zeneca and Bayer.

## Supporting information


**Table S1** International Classification of Diseases (ICD)‐10 codes used for defining clinical events between discharge and one year
**Table S2**: Discharge medications according to no switch and switch.

## Data Availability

Data Availability Statement: Data cannot be shared publicly because it was provided by the Government of Alberta under the terms of a research agreement stipulating that we do not publicly share the data. Data are available by contacting health.resdata@gov.ab.ca for researchers who meet the criteria for access to confidential information.
