# Supplementary material for: Ticagrelor or clopidogrel dual antiplatelet therapy following a pharmacoinvasive strategy in ST‐segment elevation myocardial infarction
Source: Clin Cardiol. 2021 Aug 18;44(11):1543–50. doi: 10.1002/clc.23716 (PMC8571547; doi:10.1002/clc.23716)
Supplement: Supplementary file 1 — Table S1 International Classification of Diseases (ICD)‐10 codes used for defining clinical events between discharge and one year Table S2: Discharge medications according to no switch and switch. [file CLC-44-1543-s001.docx]

Table Supplement 1: International Classification of Diseases (ICD)-10 codes used for defining clinical events between discharge and one year

| Clinical event | ICD-10 code as the ‘most responsible diagnosis’ |
| --- | --- |
| MI | I21.x, I22.x |
| Stroke | H34.1, I63, I64, I61, I60, G45 |
| ICH | I60.x, I61.x, I62.x |
| Major bleeding | K25.(0,2,4,6), K26.(0,2,4,6), K27.(0,2,4,6), K28.(0,2,4,6), K92.(0,1,2), K29.x, K31.80, K31.88, K55.8, K55.20, K62.5, K63.80, K63.88, K91.80, I60.x, I61.x, I62.x, S06.(4,5,6,7), R04.x, I85.0, I98.2 |

MI: myocardial infarction; ICH: intracranial hemorrhage.

| Table Supplement 2: Discharge medications according to no switch and switch.   \|  \| All patient  (n=1426) \| No switch  (n=1030) \| Switch  (n=396) \| P* \| \| --- \| --- \| --- \| --- \| --- \| \| ASA \| 1381 (96.8) \| 992 (96.3) \| 389 (98.2) \| 0.063 \| \| Oral anticoagulants \| 177 (12.4) \| 150 (14.6) \| 27 (6.8) \| <0.001 \| \| Warfarin \| 155 (10.9) \| 133 (12.9) \| 22 (5.6) \| <0.001 \| \| Dabigatran \| 4 (0.3) \| 4 (0.4) \| 0 (0.0) \| 0.214 \| \| Apixaban \| 4 (0.3) \| 4 (0.4) \| 0 (0.0) \| 0.214 \| \| Rivaroxaban \| 15 (1.1) \| 10 (1.0) \| 5 (1.3) \| 0.629 \| \| ACE or ARB \| 1326 (93.0) \| 955 (92.7) \| 371 (93.7) \| 0.521 \| \| ACE \| 1225 (85.9) \| 882 (85.6) \| 343 (86.6) \| 0.632 \| \| ARB \| 103 (7.2) \| 74 (7.2) \| 29 (7.3) \| 0.928 \| \| Beta Blocker \| 1336 (93.7) \| 960 (93.2) \| 376 (94.9) \| 0.225 \| \| Calcium Channel Blocker \| 6 (0.4) \| 5 (0.5) \| 1 (0.3) \| 0.543 \| \| Cholesterol Lowering Meds \| 1385 (97.1) \| 995 (96.6) \| 390 (98.5) \| 0.057 \| \| Nitroglycerin IV \| 23 (1.6) \| 20 (1.9) \| 3 (0.8) \| 0.112 \| \| Transdermal/Oral Nitroglycerin \| 4 (0.3) \| 3 (0.3) \| 1 (0.3) \| 0.901 \| \| Diuretics \| 124 (8.7) \| 97 (9.4) \| 27 (6.8) \| 0.119 \| \| Morphine \| 11 (0.8) \| 5 (0.5) \| 6 (1.5) \| 0.047 \| \| Spironolactone / Eplerenone \| 116 (8.1) \| 78 (7.6) \| 38 (9.6) \| 0.211 \|   *Comparison between no switch and switch groups.  ASA: aspirin; ACE: Angiotensin converting enzyme; ARB: angiotensin receptor blockers; IV: intravenous. |
| --- | --- | --- | --- | --- | --- | --- | --- | --- | --- | --- | --- | --- | --- | --- | --- | --- | --- | --- | --- | --- | --- | --- | --- | --- | --- | --- | --- | --- | --- | --- | --- | --- | --- | --- | --- | --- | --- | --- | --- | --- | --- | --- | --- | --- | --- | --- | --- | --- | --- | --- | --- | --- | --- | --- | --- | --- | --- | --- | --- | --- | --- | --- | --- | --- | --- | --- | --- | --- | --- | --- | --- | --- | --- | --- | --- | --- | --- | --- | --- | --- | --- | --- | --- | --- | --- | --- | --- | --- | --- | --- |
